# Changes in gray matter volume and functional connectivity in dementia with Lewy bodies compared to Alzheimer’s disease and normal aging: implications for fluctuations

**DOI:** 10.1186/s13195-019-0575-z

**Published:** 2020-01-06

**Authors:** Eléna Chabran, Vincent Noblet, Paulo Loureiro de Sousa, Catherine Demuynck, Nathalie Philippi, Catherine Mutter, Pierre Anthony, Catherine Martin-Hunyadi, Benjamin Cretin, Frédéric Blanc

**Affiliations:** 1 ICube Laboratory UMR 7357 and FMTS (Fédération de Médecine Translationnelle de Strasbourg), Team IMIS, University of Strasbourg and CNRS, Strasbourg, France; 2 CM2R (Research and Resources Memory Centre), Geriatrics Department, University Hospitals of Strasbourg, Geriatric Day Hospital and Neuropsychology Unit, Strasbourg, France; 3 INSERM Centre d’Investigation Clinique 1434, University Hospitals of Strasbourg, Strasbourg, France; 4 General Hospital Centre, Geriatrics Department, CM2R, Geriatric Day Hospital, Colmar, France

**Keywords:** Dementia with Lewy bodies, Fluctuations, MRI, Functional connectivity, Voxel-based morphometry, Salience network

## Abstract

**Background:**

Fluctuations are one of the core clinical features characterizing dementia with Lewy bodies (DLB). They represent a determining factor for its diagnosis and strongly impact the quality of life of patients and their caregivers. However, the neural correlates of this complex symptom remain poorly understood. This study aimed to investigate the structural and functional changes in DLB patients, compared to Alzheimer’s disease (AD) patients and healthy elderly subjects, and their potential links with fluctuations.

**Methods:**

Structural and resting-state functional MRI data were collected from 92 DLB patients, 70 AD patients, and 22 control subjects, who also underwent a detailed clinical examination including the Mayo Clinic Fluctuation Scale. Gray matter volume changes were analyzed using whole-brain voxel-based morphometry, and resting-state functional connectivity was investigated using a seed-based analysis, with regions of interest corresponding to the main nodes of the salience network (SN), frontoparietal network (FPN), dorsal attention network (DAN), and default mode network (DMN).

**Results:**

At the structural level, fluctuation scores in DLB patients did not relate to the atrophy of insular, temporal, and frontal regions typically found in this pathology, but instead showed a weak correlation with more subtle volume reductions in different regions of the cholinergic system. At the functional level, the DLB group was characterized by a decreased connectivity within the SN and attentional networks, while the AD group showed decreases within the SN and DMN. In addition, higher fluctuation scores in DLB patients were correlated to a greater connectivity of the SN with the DAN and left thalamus, along with a decreased connectivity between the SN and DMN, and between the right thalamus and both the FPN and DMN.

**Conclusions:**

Functional connectivity changes, rather than significant gray matter loss, could play an important role in the emergence of fluctuations in DLB. Notably, fluctuations in DLB patients appeared to be related to a disturbed external functional connectivity of the SN, which may lead to less relevant transitions between different cognitive states in response to internal and environmental stimuli. Our results also suggest that the thalamus could be a key region for the occurrence of this symptom.

**Electronic supplementary material:**

The online version of this article (10.1186/s13195-019-0575-z) contains supplementary material, which is available to authorized users.

## Background

Dementia with Lewy bodies (DLB) is the second most common cognitive neurodegenerative disorder after Alzheimer’s disease (AD), accounting for 15–20% of pathologically diagnosed dementia cases [1]. The core clinical features characterizing DLB, along with dementia, are cognitive fluctuations, recurrent visual hallucinations, rapid eye movement sleep behavior disorder (RBD) and spontaneous cardinal features of parkinsonism [1]. Of these symptoms, cognitive fluctuations appear to be both the most typical and the least understood. Defined as spontaneous alterations in cognition with pronounced variations in attention and alertness, they occur in 80–90% of DLB patients [1, 2] and have a significant impact on patients’ autonomy and quality of life [3–5]. In daily life, they result in substantial changes in patients’ mental status and behavior over time, alternating between periods of poorer task performance, incoherent speech and behavior, excessive daytime somnolence and episodes of altered consciousness (described by caregivers as sudden “switching off”), and periods of greater lucidity and cognitive performance [1, 6, 7]. Their periodicity and duration are highly variable even within the same person, ranging from seconds or minutes, to months. Clinically, fluctuations in DLB patients must be distinguished from the mild, day-to-day fluctuations commonly observed in various forms of dementia. They were shown to have a greater prevalence and severity than those observed in AD and vascular dementia [2, 7, 8], and to be qualitatively distinct from fluctuations in AD patients, which rather consist in episodes of memory failure [9]. Furthermore, fluctuations in DLB patients seem to be internally driven, while those in AD patients may be more related to environmental triggers [9].

Despite fluctuations being a major symptom of DLB, their neural bases remain unclear. First, no consistent structural brain changes have been related to cognitive fluctuations, although a few studies have investigated this question. In a study combining diffusion tensor MRI and proton MR spectroscopy, Delli Pizzi et al. [10] reported a cholinergic imbalance in the right thalamus in DLB patients compared to controls and AD patients, along with microstructural alterations in bilateral thalamic regions projecting to the frontal cortex. However, only cholinergic measures correlated with the presence and severity of cognitive fluctuations, suggesting that neurochemical changes in this region could be more relevant to the physiopathology of fluctuations than microstructural alterations. More recently, significant atrophy was also found in the left pulvinar and ventral lateral nucleus regions of the thalamus in DLB patients and appeared to be associated with impaired attentional function [11]. Underlining the link between attentional impairment and cognitive fluctuations, the authors thus suggested a potential role of thalamic atrophy in the latter symptom. Lastly, a volumetric study focusing on the substantia innominata (SI) showed a significant negative association between the severity of cognitive fluctuations and gray matter volume in the right SI in DLB patients [12]. Overall, current findings do not allow clear conclusions on the structural correlates of this symptom but provide some clues towards a potential involvement of subcortical structures.

At the same time, an increasing focus is put on functional aspects, recent studies assuming that cognitive fluctuations are more likely to arise from functional network disturbances rather than overt structural abnormalities [13, 14]. Electroencephalographic data showed that the frequency and severity of cognitive fluctuations were correlated with an increase and abnormal variability of posterior slow wave activity in DLB patients compared to AD patients [4, 15–17]. Similar posterior disturbances were observed using single-photon emission computed tomography (SPECT): cognitive fluctuations in DLB patients appeared to be associated with decreased inferior occipital perfusion and increased thalamic perfusion in one study [18] and with decreased perfusion in bilateral posterior parietal regions, covariant with increased perfusion in motor regions, in another [13]. In terms of functional connectivity, a significant association was found between greater fluctuations and a reduced connectivity between the right middle frontal gyrus and the right lateral parietal cortex in DLB patients [19]. Peraza et al. [14] also reported a positive correlation between alterations within the left frontoparietal network and the severity and frequency of cognitive fluctuations in DLB. According to these results, functional perturbations related to fluctuations thus seem to involve widely distributed networks rather than one particular brain region.

The present study sought to complement and clarify current knowledge on the neural bases of fluctuations in DLB patients, by assessing both structural and functional aspects. Our aims were, first, to investigate changes in gray matter volume and functional connectivity in DLB patients compared to AD patients and healthy elderly subjects and, second, to assess more directly whether these structural and functional measures correlate with fluctuation scores in DLB patients. Since, in most of the previous studies, morphometric analyses were limited to a few a priori defined regions of interest, and functional connectivity analyses mainly focused on within-network changes, we aimed to provide a broader analysis framework by conducting our structural analysis on the whole brain and examining both within- and between-network functional connectivity in a set of well-described resting-state networks.

## Methods

### Participants

A total of 184 subjects participated in the study. Ninety-two DLB patients and 70 AD patients were recruited through member teams of the Centre Mémoire de Ressources et de Recherche (CM2R) Alsace, France (i.e., the Geriatrics and Neurology Departments of the University Hospital of Strasbourg [HUS] and the Geriatrics Department of the General Hospital Center of Colmar). Additionally, 22 elderly healthy subjects were recruited through the Clinical Investigation Centre and CM2R of HUS. The study was approved by the Est-IV ethics committee (CPP Est-IV, Strasbourg), and all participants provided written informed consent.

To be included, the subjects had to be over the age of 50 years and be native speakers of French. Exclusion criteria were as follows: MRI contraindications, alcohol or substance abuse, sensory or motor disability, additional neurological or psychiatric conditions that could explain the symptoms, significant focal cerebral lesions shown on brain imaging, and co-occurrence of DLB and AD.

DLB and AD patients were diagnosed by experienced neurologists and geriatricians on the basis of the McKeith [2] and the Dubois [20] criteria, and the control participants were examined in the same way as patients to exclude any occult mild cognitive impairment or dementia cases. The potential presence of medial temporal atrophy was assessed using the Scheltens visual rating scale on brain MRI scans for the three groups. All participants underwent detailed clinical and neuropsychological evaluations, including the Mini-Mental State Examination (MMSE) and the Mayo Clinic Fluctuation Scale [6]. For a comprehensive description of the different neuropsychological assessments, see Kemp et al. [21].

We excluded 25 participants (13 DLB and 12 AD patients) from the functional analysis for reasons of missing data (*n* = 12), image artifacts (*n* = 9), and to match the two dementia groups in terms of mean MMSE (*n* = 4).

### Demographic and cognitive measures

Statistical analyses of demographic and cognitive measures were performed using GraphPad Prism software (GraphPad Software, Inc., San Diego, CA). Normality of distribution of the variables was tested using the Shapiro-Wilk test and by visual inspection of quantile-quantile plots, and homogeneity of variances across groups was assessed using Levene’s test. Where appropriate, between-group differences in continuous data were evaluated using either the non-parametric Kruskal-Wallis test with Dunn’s post hoc test or a one-way ANOVA with Tukey’s post hoc test. For categorical data, we used the chi-square test. Results were regarded as significant at *p* < 0.01.

### MRI data acquisition

Imaging was performed using a Siemens Verio 3T scanner equipped with a 32-channel head coil (Siemens, Erlangen, Germany). A concomitant resting-state blood oxygen level-dependent (BOLD) and arterial spin-labeling sequence was used to acquire 121 whole-brain T2*-weighted (gradient echo) echo planar images. The parameters were as follows: repetition time = 3 s; flip angle = 90°; echo time = 21 ms; inversion time 1 = 600 ms; inversion time 2 = 1325.1 ms; field of view = 152 × 256 × 112 mm; 4-mm isotropic voxels. The first volume was intended for arterial spin-labeling measurement; thus, it was not considered for BOLD analysis. One whole-brain T1-weighted image was also collected within the same session, using a 3D magnetization-prepared rapid gradient-echo (MPRAGE) sequence. The parameters were as follows: repetition time = 1.9 s; flip angle = 9°; echo time = 2.53 ms; inversion time = 900 ms; field of view = 192 × 192 × 176 mm; 1-mm isotropic voxels.

### MRI data preprocessing

Images from each subject were preprocessed using the Statistical Parametric Mapping 12 package (SPM12, The Wellcome Trust Centre for Neuroimaging, London, UK).

#### Structural images

The anatomical data set was first visually inspected to check for potential artifacts or anatomical abnormalities and then spatially preprocessed using standard procedures [22]. All T1 structural images were segmented and bias corrected using an extension of the unified segmentation procedure [23] including six classes of tissue. The DARTEL approach was then used to build a study-specific template and to spatially normalize all segmented images to the Montreal Neurological Institute (MNI) space. Lastly, gray matter images were modulated to preserve the total amount of gray matter from the original data and smoothed with a 8-mm full-width at half-maximum Gaussian kernel.

#### Functional images

Functional images were preprocessed with the following steps: low-pass filtering at 0.112 Hz to remove arterial spin-labeling frequencies; slice-timing correction; rigid body registration and B0 field inhomogeneity correction; coregistration to the T1-weighted anatomical image; spatial normalization to Montreal Neurological Institute space using the DARTEL approach with an 8-mm full-width at half-maximum Gaussian kernel. No modulation was applied during the normalization procedure. We ensured that there were no between-group differences in the total amount of head motion (corresponding to the maximum absolute frame-wise displacement across scans), using ANOVA (F(2, 156) = 0.34, *p* = 0.71). Finally, a denoising step using the aCompCor method [24] including cerebrospinal fluid, white matter, and motion parameters was conducted to remove residual unwanted motion and physiological and artefactual effects from the BOLD signal, prior to connectivity analyses.

### Statistical analysis

#### Voxel-based morphometry (VBM) analysis

##### Group comparisons

Between-group differences in gray matter volume were assessed using the SPM12 General Linear Model based on Gaussian random field theory [25]. Age, gender, and total intracranial volume were included in the design matrix as covariates of no interest. Statistical significance was set at a false discovery rate (FDR)-corrected threshold of *p* < 0.001 at the voxel level.

##### Association between VBM measures and clinical variables

A multiple regression analysis was performed to examine the effect of gray matter volume on fluctuation scores in the DLB group separately. Variables entered into the model along with the dependent variable included the fluctuation scores, as a covariate of interest, and age, sex, and total intracranial volume, as covariates of no interest. Results were reported when significant at an uncorrected threshold of *p* < 0.05 at the voxel level.

#### Resting-state functional connectivity analysis

##### ROI-to-ROI analysis

**Fig. 1:**
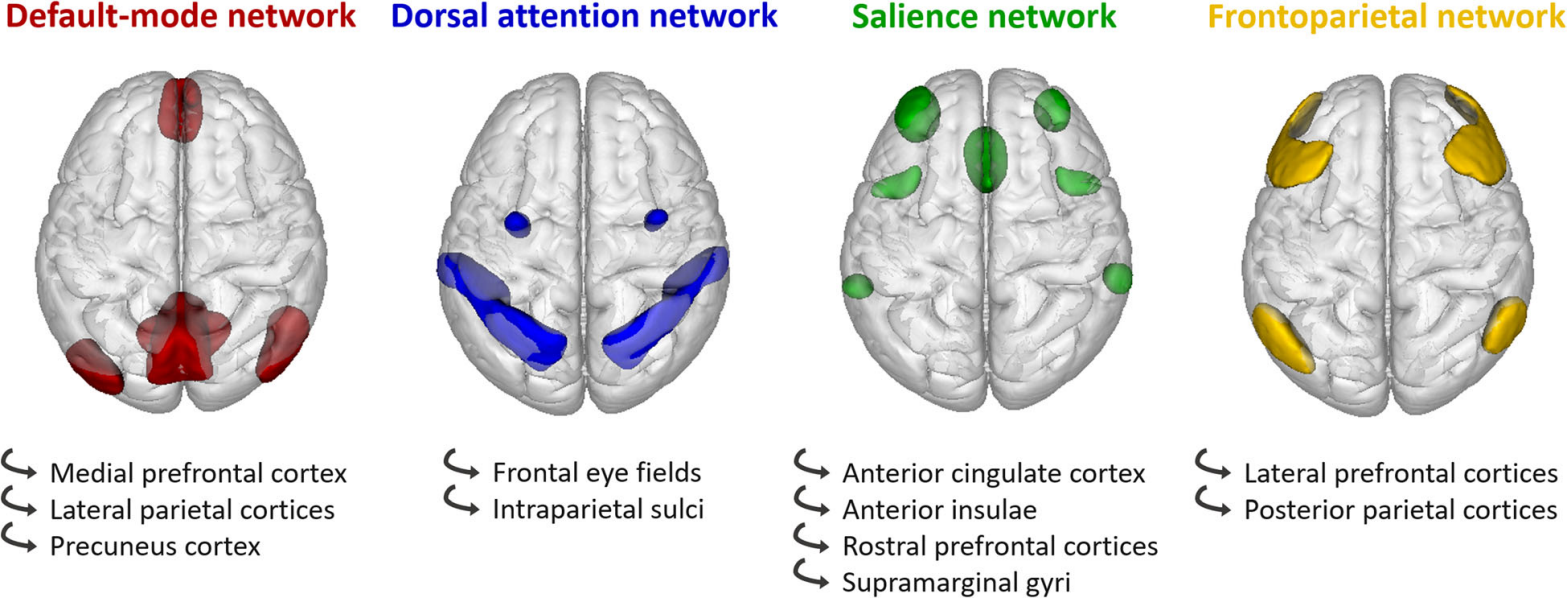
Regions of interest used in the functional connectivity analysis, superimposed on a three-dimensional brain template (superior view)

Seed-based functional connectivity analyses were performed using the Conn toolbox [26]. Twenty-two regions of interest (ROIs) corresponding to the main nodes of the salience network (SN), frontoparietal network (FPN), dorsal attention network (DAN), and default mode network (DMN) were selected from the “networks atlas” implemented in the Conn toolbox, which was obtained from an independent component analysis (ICA) including 497 subjects from the Human Connectome Project dataset [26]. Additionally, two other ROIs were defined based on the results of the VBM analysis, corresponding to brain regions where gray matter volume was correlated to fluctuation scores in DLB patients. They were added in the connectivity analysis using the Harvard-Oxford Atlas [27] implemented in the Conn toolbox. See Fig. 1 and Additional file 1: Table S1.

At the first level of the analysis, individual ROI-to-ROI functional connectivity matrices were generated by computing bivariate Pearson’s correlation measures between the mean BOLD signal time courses of each pair of ROIs. Each participant’s motion parameters obtained during the preprocessing were added as a covariate of no interest. A Fisher transformation was then applied to the correlation coefficients to improve normality assumptions of the subsequent, second-level analyses.

In a next step, the individual matrices were entered into a second-level general linear model, with age and gender as covariates of no interest, to allow between-group comparisons. The results were reported only when surviving a false discovery rate (FDR) corrected threshold of *p* < 0.05 at the seed level.

##### Mean within- and between-network connectivity

In addition, mean within-network and between-network connectivity values were computed in the second level of the analysis, to obtain more summary measures of the functional changes in the different groups of participants. The mean within-network connectivity was calculated as an average of the connectivity values between all ROIs within a network, while the mean between-network connectivity corresponded to an average of the connectivity values between all possible ROI pairs in two chosen networks.

##### Association between connectivity measures and clinical variables

Multiple regression analyses were also performed to examine the effect of functional connectivity on fluctuation scores, in the DLB group separately. Variables entered into the model along with the dependent variable included the fluctuation scores as a covariate of interest, and age and gender as covariates of no interest. Results were reported when significant at an uncorrected threshold of *p* < 0.05 at the seed level.

## Results

### Demographic and cognitive measures

Demographic and cognitive data of the participants are summarized in Table 1. In the VBM cohort, the AD group was older than the control and DLB groups, while the latter were comparable in age. In the cohort used for the functional analysis (which was the same cohort as for the VBM analysis but with 25 fewer participants), the AD group was older than the control group but not the DLB group, and the latter were comparable in age. In both cohorts, the two dementia groups had lower MMSE scores than the control group, but did not differ from each other for this variable. As expected, the DLB group had higher fluctuation scores than both other groups. Finally, all groups were comparable for gender.

**Table 1:** Demographics and fluctuation scores of the cohorts

A	HC (*n* = 22)	DLB (*n* = 92)	AD (*n* = 70)	Between-group differences
Gender (m/f)	11/11	39/40	26/32	*χ*^2^ = 0.42, *p* = 0.81
Age	66.5 ± 7.8	70.1 ± 9.4	74.4 ± 8.3	AD vs HC: *p* = 0.0010 AD vs DLB: *p* = 0.0084 DLB vs HC: NS
MMSE	28.9 ± 0.9	25.6 ± 4.0	23.8 ± 3.8	DLB vs HC: *p* = 0.0001 AD vs HC: *p* < 0.0001 DLB vs AD: NS
MCFS	0.3 ± 0.6	1.9 ± 1.2	0.7 ± 1.0	DLB vs HC: *p* < 0.0001 DLB vs AD: *p* < 0.0001 AD vs HC: NS
B	HC (*n* = 22)	DLB (*n* = 79)	AD (*n* = 58)	Between-group differences
Gender (m/f)	11/11	39/40	26/32	*χ*^2^ = 0.33, *p* = 0.85
Age	66.5 ± 7.8	70.3 ± 9.5	73.7 ± 8.3	AD vs HC: *p* = 0.0045 DLB vs HC: NS DLB vs AD: NS
MMSE	28.9 ± 0.9	25.8 ± 3.8	24.4 ± 3.2	DLB vs HC: *p* = 0.0002 AD vs HC: *p* < 0.0001 DLB vs AD: NS
MCFS	0.3 ± 0.6	1.8 ± 1.2	0.6 ± 0.9	DLB vs HC: *p* < 0.0001 DLB vs AD: *p* < 0.0001 AD vs HC: NS

(A) Cohort of the voxel-based morphometry analysis. (B) Cohort of the functional connectivity analysis. Data are presented as mean ± SD except where noted. *Abbreviations*: *AD* Alzheimer’s disease, *DLB* dementia with Lewy bodies, *HC* healthy controls, *f* female, *m* male, *MCFS* Mayo Clinic Fluctuation Scale, *MMSE* Mini-Mental State Examination, *NS* non-significant

### VBM analysis

#### Group comparisons

**Fig. 2:**
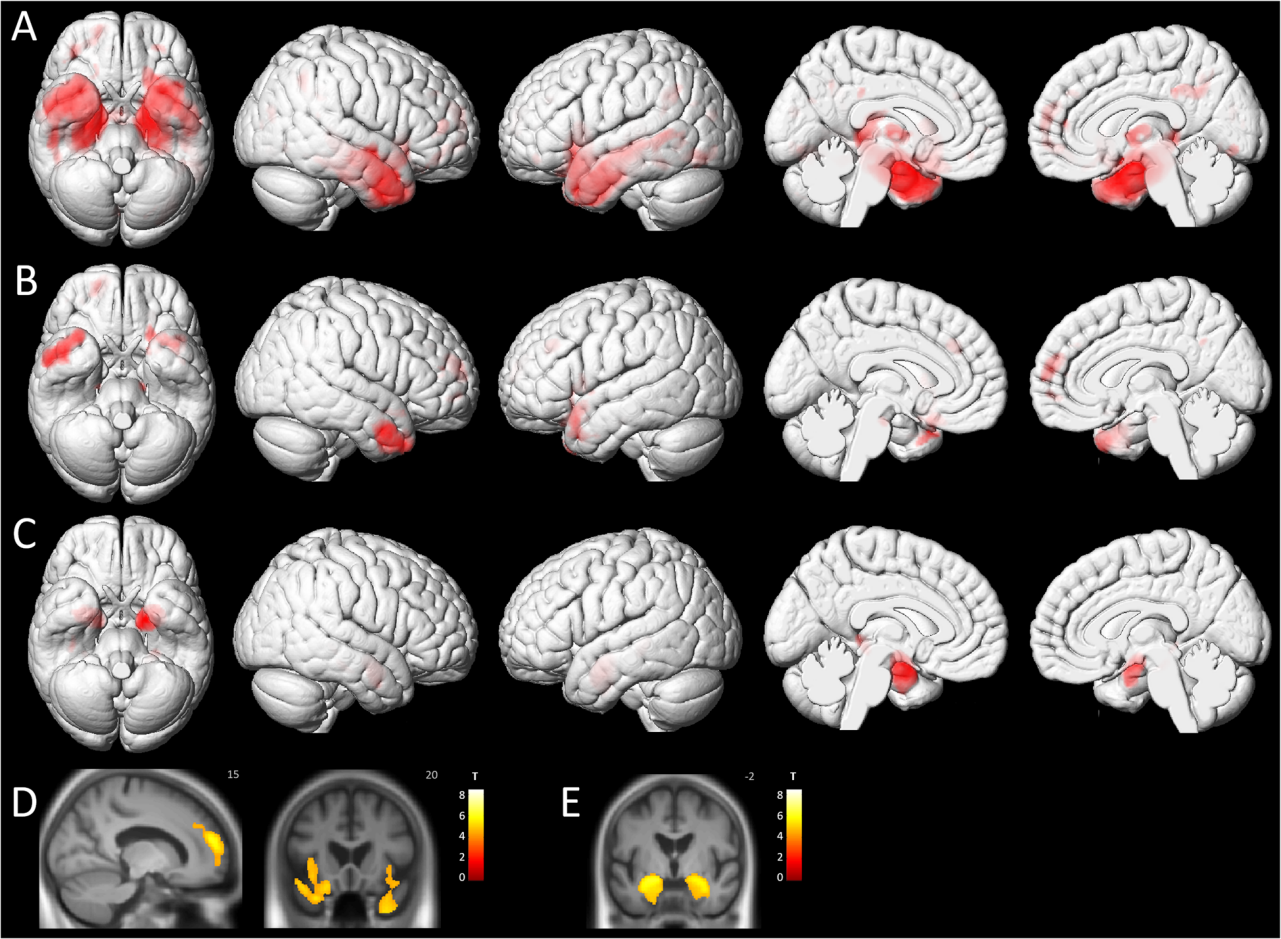
Patterns of significant voxel-wise gray matter loss in the three groups. **a** AD patients compared to controls, **b** DLB patients compared to controls, and **c** AD patients compared to DLB patients, superimposed on three-dimensional MNI surface renders. **d** DLB patients compared to controls and **e** AD patients compared to DLB patients, superimposed on a mean T1 image (neurological orientation). All results are represented at *p* < 0.001 (FDR-corrected) at the voxel level

Compared with the control group, the AD group showed a widespread pattern of atrophy involving mainly the medial temporal lobe. The DLB group differed from the controls by showing more focal patterns of gray matter loss, involving the temporal lobes, the insulae, and the frontal lobes. When comparing the AD and DLB groups, we observed a greater gray matter loss in the medial temporal lobe (including the parahippocampal gyrus, the hippocampus and the amygdala) in AD patients. No regions showed greater atrophy in the control group than in the dementia groups, nor in the DLB group than in the AD group (see Fig. 2).

#### Association between VBM measures and fluctuation scores

In the DLB group (see Table 2), we found a negative correlation between patients’ fluctuation scores and gray matter volume in clusters including the left inferior parietal lobule, the left and right cerebellum, the midbrain, the right middle orbitofrontal gyrus, and the left superior orbitofrontal gyrus. When raising the threshold to *p* < 0.005, this negative correlation also appeared in the right thalamus.

Among these regions, the midbrain and the thalamus were added as complementary ROIs for the subsequent functional connectivity analysis. The left inferior parietal gyrus and the prefrontal cortex did not need to be added as they already corresponded to nodes of the selected functional networks.

We did not find any cluster where gray matter volume was positively correlated to patients’ fluctuation scores.

**Table 2:** Location and peak significance of clusters where gray matter volume was negatively correlated with fluctuation scores in the DLB group

Anatomical region	Peak level (*p*_unc._)	Extent (k)	*t*	*z*	MNI coordinates (*x*,*y*,*z*)		
Left inferior parietal lobule	*p* < 0.0001	141	4.63	4.36	− 45	− 45	52.5
Left cerebellum	*p* < 0.0001	165	4	3.82	− 9	− 52	− 32
Right cerebellum	*p* < 0.0001	1212	3.97	3.79	42	− 54	26
Midbrain	*p* < 0.0001	35	3.47	3.35	−19.5	− 16.5	− 12
Right middle frontal gyrus (orbital part)	*p* = 0.0008	15	3.27	3.17	46	51	− 4.5
Left superior frontal gyrus (orbital part)	*p* = 0.0009	9	3.24	3.14	− 10.5	42	− 24
Right thalamus	*p* = 0.0027	186	2.86	2.79	3	− 8	4

### Functional connectivity analysis

#### ROI-to-ROI analysis

**Fig. 3:**
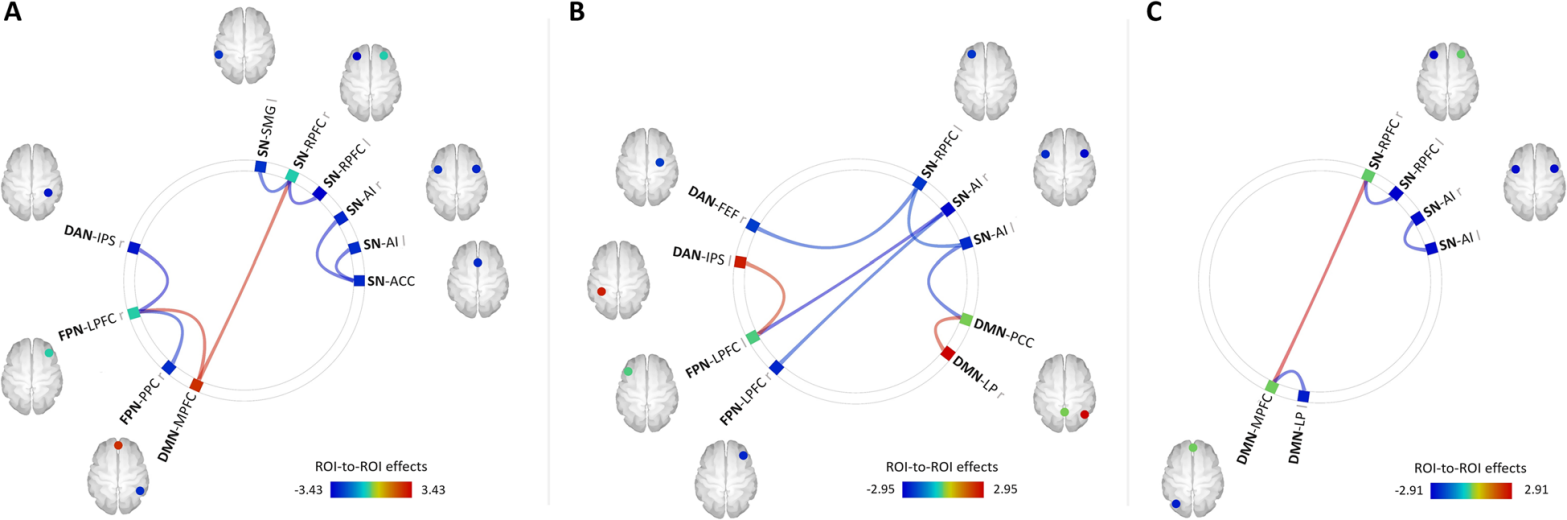
Between-group differences in ROI-to-ROI functional connectivity. **a** DLB > controls, **b** DLB > AD, and **c** AD > controls. *Abbreviations*: ACC, anterior cingulate cortex; AI, anterior insula; DAN, dorsal attention network; DMN, default-mode network; FEF, frontal eye field; FPN, frontoparietal network; IPS, intraparietal sulcus; l, left; LP, lateral parietal cortex; LPFC, lateral prefrontal cortex; MPFC, medial prefrontal cortex; PCC, precuneal cortex; PPC, posterior parietal cortex; r, right; RPFC, rostral prefrontal cortex; SN, salience network; SMG, supramarginal gyrus. The results are represented respectively at **a** *p*_FWE_ < 0.05, **b** *p*_unc_ < 0.01, and Figure S1 *p*_unc_ < 0.05, at the ROI level

First, the DLB group showed a significant decrease in functional connectivity between a number of ROIs within the SN, compared to the control group (see Fig. 3). Similar decreases were found between ROIs within the FPN (right lateral prefrontal cortex [LPFC] and right posterior parietal cortex [PPC]) and between ROIs of the FPN and the DAN (right LPFC and right intraparietal sulcus [IPS], respectively). In contrast, a significantly increased functional connectivity was observed between the medial prefrontal cortex (MPFC) of the DMN and both the right rostral prefrontal cortex (RPFC) of the SN and the right LPFC of the FPN, in DLB patients compared to controls.

No significant differences were found between the AD and control groups or between the DLB and AD groups at the set FDR-corrected threshold. At an uncorrected threshold of *p* < 0.05, AD patients showed a connectivity decrease between several ROIs within the SN and within the DMN, and a higher connectivity between the MPFC of the DMN and the right RPFC of the SN, compared to the control group. When comparing the AD and DLB groups (*p* < 0.01 uncorrected), DLB patients had a weaker connectivity between two ROIs within the SN (left RPFC and left anterior insula [AI]), and between ROIs of this network and ROIs of the DAN (right frontal eye field [FEF]), FPN (left and right LPFC), and DMN (precuneal cortex [PCC]). Conversely, they showed a higher connectivity than AD patients between two ROIs within the DMN (right lateral parietal cortex [LP] and PCC) and between the left IPS of the DAN and the left LPFC of the FPN (see Fig. 3).

#### Mean within- and between-network connectivity

The DLB group and AD group showed a weaker mean functional connectivity within the SN compared to the control group (*T*_154_ = 2.96, *p* = 0.0035 and *T*_154_ = 2.04, *p* = 0.0430, respectively). No other within-network and no between-network connectivity differences were found among the three groups.

#### Association between FC measures and fluctuation scores

**Fig. 4:**
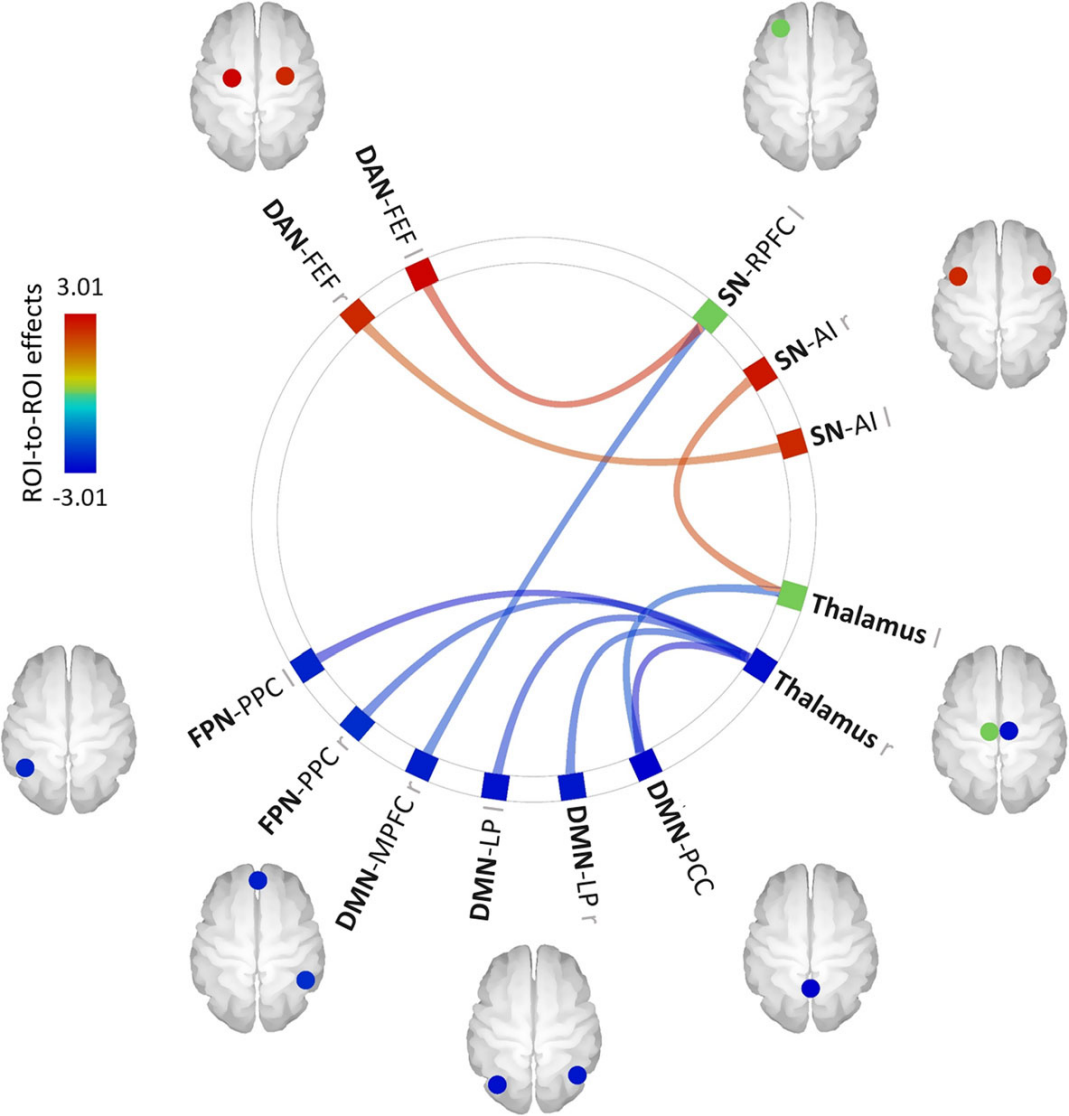
Effect of fluctuation scores on functional connectivity in the DLB group. *Abbreviations*: AI, anterior insula; DAN, dorsal attention network; DMN, default-mode network; FEF, frontal eye field; FPN, frontoparietal network; l, left; LP, lateral parietal cortex; MPFC, medial prefrontal cortex; PCC, precuneus cortex; PPC, posterior parietal cortex; r, right; RPFC, rostral prefrontal cortex; SN, salience network. The results are represented at *p*_unc_ < 0.05 at the ROI level

In the DLB group, we found a positive correlation between patients’ fluctuation scores and functional connectivity for three pairs of ROIs: left SN-RPFC and left DAN-FEF, left SN-AI and right DAN-FEF, right SN-AI and left thalamus. Fluctuation scores were also negatively correlated to the functional connectivity between the left SAL-RPFC and the DMN-MPFC, between the right thalamus and all the ROIs of the DMN (MPFC, left and right LP, PCC), between the right thalamus and two ROIs of the FPN (left and right PPC), as well as between the left thalamus and the DMN-PCC (see Fig. 4).

## Discussion

In the present study, we aimed to investigate the structural and functional changes in DLB compared with AD and normal aging and to examine their potential role in the emergence of the fluctuations characterizing these patients.

With regard to structural aspects, the results of group comparisons performed in our VBM analysis were in agreement with the existing literature. In the AD group compared to the control group, we found a typical pattern of medial temporal atrophy, as consistently described in previous studies [28]. In contrast, gray matter loss in the DLB group compared to controls was less extensive and consisted of focal bilateral clusters in the temporal lobes, insulae, and frontal lobes. The changes observed in temporal and frontal regions were consistent with the results of several earlier VBM studies [29, 30]. Similarly, diminished gray matter volumes in bilateral insulae were previously reported in DLB patients at both the dementia [31] and prodromal [32, 33] stages. The insular cortex was also shown to be particularly vulnerable to α-synuclein pathology in these patients [34]. Unlike some previous studies [35–37], we did not find any significant gray matter loss in subcortical structures in the DLB group compared to the control group. This could be explained by the fact that the group comparisons in most of these studies were performed on chosen segmented subcortical structures rather than on the whole brain gray matter. Another possible explanation to this result is that our DLB patients were at early stages of the disease (prodromal and mild dementia stages), with a mean group MMSE of 25.6 indicating a very mild cognitive decline. Previous studies did not show subcortical gray matter loss in DLB patients at the prodromal stage [32, 33], so it is possible that changes in these regions occur later in the disease course. To further explore the potential subcortical gray matter changes in our DLB group, we evaluated the differences between the DLB and control subjects with a more permissive threshold of *p* < 0.0001 (uncorrected for multiple comparisons). The results are shown in Additional file 2: Figure S1. Our early-stage DLB patients showed trends of decreased gray matter volume in the thalamus and in the brainstem. These slight changes, which are not sufficiently marked to reach significance with an FDR correction, are consistent with previous studies showing atrophy in the same regions in these patients at the dementia stages [35, 36, 38] and tend to support the hypothesis of a later occurrence of subcortical volume loss in DLB. Furthermore, as pointed out in a recent review [39], the most significant subcortical changes reported in DLB patients in the literature relate to functional rather than structural imaging data. Finally, the comparison between the AD and DLB groups revealed a relative bilateral preservation of the medial temporal lobe structures in the latter, in line with a number of existing data reviewed by Surendranathan [40].

Among these results, gray matter losses found in the insulae and frontal regions in the DLB group may be relevant with regard to fluctuations. The insula is a complex cortical region participating in a wide range of cognitive, emotional, somatosensory, and visceral functions [41], and its anterior division forms a major node of the SN. Considering the critical role of the SN for the switching between default-mode and task-positive networks [42] and the well-known involvement of frontal regions in attentional-executive processes, such atrophic patterns could potentially participate in fluctuations in DLB patients.

However, when investigating more directly the correlation between patients’ fluctuation scores and gray matter volume in the DLB group, we found a weak negative correlation in the left inferior parietal lobule, the left and right cerebellum, the midbrain, the bilateral prefrontal cortex, and to a lesser extent, the right thalamus. These regions did not correspond to those showing gray matter loss in DLB patients compared to control subjects, which suggests that the relative atrophy of frontal and insular cortices may not be directly involved in the occurrence of fluctuations. Moreover, the effect of fluctuation scores on regional gray matter volume seemed to follow a particular pattern, as the corresponding brain regions were part of the cholinergic system. This system plays an important role in attention and conscious awareness [43, 44] and was shown to be more affected in DLB than in AD [45, 46]. The disturbances identified in DLB patients notably include deficits in ChAT [47] and AChE [48] and changes in nicotinic and muscarinic receptors density [49–51] compared to AD patients and healthy elderly subjects. Several studies reviewed by Aarsland et al. [52] also demonstrated that cholinesterase inhibitors have a positive effect on cognition and neuropsychiatric symptoms in DLB patients, including fluctuating attention, unresponsiveness, and daytime somnolence [53]. Similarly, neuropathological analyses comparing fluctuating and non-fluctuating DLB patients reported significant cholinergic impairments in thalamic areas in the former [54]. Taken together, these findings suggest that cholinergic impairments may be a determinant factor in the etiology of cognitive fluctuations. In the abovementioned regions where gray matter volume was found to be negatively correlated to fluctuation scores, but which are not significantly atrophied in DLB patients compared to controls, it is possible that cholinergic synaptic dysfunction may cause microstructural impairments (such as a reduced synaptic density) without frank neuronal loss, thus only leading to subtle volume reductions. Indeed, other neurotransmitter systems involved in attention and arousal, such as noradrenergic or dopaminergic systems, suffer neuronal loss in DLB [55, 56] and could therefore also contribute to a lesser degree to the pathogenesis of this symptom.

Globally, the results of our VBM analysis thus suggest that fluctuations might be more related to microstructural (i.e., slight volume reductions secondary to a loss of synapses) or functional changes, rather than macrostructural modifications.

To investigate the potential spatial correspondence between structural and functional disturbances related to fluctuations in the DLB group, the thalami and midbrain were added as complementary ROIs for the subsequent functional connectivity analysis (the inferior parietal and prefrontal regions did not need to be added as they already corresponded to key nodes of the preselected functional networks).

The second part of our study focused on functional connectivity aspects. At the network level, when compared to the control group, the DLB patients showed significant decreases in functional connectivity within the SN and within the FPN, and between ROIs of the FPN and DAN. In contrast, they showed an increased connectivity between ROIs of the DMN and ROIs of both the SN and the FPN. In the AD group, the changes relative to the control group were globally weaker than those distinguishing the DLB group from the control group. They showed a decreased functional connectivity within the SN but also within the DMN, along with an increased connectivity between ROIs from those two networks. Finally, when comparing directly the two dementia groups, DLB patients showed a weaker ROI-to-ROI functional connectivity than AD patients within the SN, and between this network and the DAN, the DMN and the FPN. Conversely, the DLB group had a stronger connectivity than the AD group within the DMN, and between ROIs of the DAN and FPN. Additional measurements confirmed a weaker mean connectivity within the SN in the DLB and AD groups compared with the control group.

These results tend to identify specific patterns of functional connectivity disturbances in DLB and AD. Both groups showed a decreased connectivity within the SN compared to healthy elderly subjects, which was accompanied by an increased connectivity between the right RPFC of the SN and the MPFC of the DMN. This increase could constitute a compensation process, in order to maintain an effective coupling between the two networks despite the functional disruptions in the SN. In the DLB group, the increased connectivity between the FPN-LPFC and the DMN-MPFC could similarly be an attempt to compensate for the connectivity disturbances within and between attentional networks. But beyond these similarities, the two groups also revealed distinct profiles of impairments, as SN disturbances appeared to co-occur primarily with attentional network disruptions in the DLB group, while they rather coincided with DMN disruptions in the AD group. In AD patients, this decrease in DMN functional connectivity is a consistently reported feature in previous studies, irrespective of the analytical approach used [57]. Regarding the SN, the literature is less consistent: some authors reported an increased functional connectivity in this network in AD compared to normal aging [58, 59], while others found it was decreased [60, 61]. This could be explained by methodological differences between the analyses (ICA versus seed-based approaches). However, Brier et al. [62] provided another possible explanation by showing in a larger cohort (*n* = 124) that functional connectivity within the SN was increased in AD patients at a very early stage of the disease (clinical dementia rating [CDR] 0.5), but then decreased at a later stage (CDR 1).

In DLB, our findings are similar to those of Lowther et al. [63], who found a decreased connectivity in the salience and executive control networks compared with AD patients and healthy subjects. The fact that we found decreases specifically within the FPN and the SN and between the FPN and the DAN, may provide some clues about the occurrence of fluctuations and attention deficits [1, 3] in DLB patients, considering the particular interaction between these networks. The FPN and DAN show high activity during externally directed attentional focus and are anticorrelated with the DMN, which is activated during unfocused, internally focused, or exploratory states [64, 65]. The coupling of these networks, which allows an effective switching between distinct attentional states in response to both internal and environmental stimuli, was shown to be triggered by the activity of the SN [42, 66]. The functional connectivity disruptions that we observed in the DLB group may lead to disturbed transitions from one network configuration (or cognitive state) to another and could thus participate in the etiology of fluctuations.

The multiple regression analysis revealed that the severity of fluctuations in DLB patients was positively correlated to the functional connectivity of the SN with the DAN and the left thalamus and negatively correlated with the connectivity between the SN and the DMN and between the right thalamus and both the FPN and the DMN.

First, this suggests that fluctuations could arise from an imbalance of the external connectivity of the SN, maybe triggered primarily by the disconnections between the different ROIs within this network, as observed in the group comparisons. The negative correlation between fluctuation scores and functional connectivity between the SN and DMN is striking because it involves the RPFC of the SN and the MPFC of the DMN, a pair of ROIs showing a hyperconnectivity (but with a different lateralization) in DLB patients compared to healthy subjects, which we interpreted as a compensation process. In agreement with this hypothesis, greater fluctuations in DLB patients may thus be linked to a failure of this compensation. Second, these findings tend to confirm the involvement of thalamic functional disturbances in the etiology of fluctuations in DLB patients, as suggested by previous studies [10, 18]. The thalamus has strong functional connections with cortical nodes of the SN, especially the anterior insula hub, and participates with it in a large-scale network integrating interoceptive inputs with cognitive inputs from other networks and generating viscero-autonomic, emotional, and cognitive processes in response to salient stimuli [67, 68]. Moreover, the functional and structural connectivity of the thalamus with the DMN [69, 70] and the frontoparietal regions [71] was shown to be crucial for consciousness. The negative correlation between thalamic functional connectivity and fluctuation scores in the DLB group therefore seems concordant with the clinical characteristics of this symptom. Furthermore, this correlation could also be consistent with the results of our VBM analysis showing a negative correlation between fluctuation scores and gray matter volume in several cholinergic regions. Indeed, as mentioned by Peraza et al. [72], loss of cholinergic function may impair the ability of the cholinergic system to inhibit intracortical short-range functional connections, which is the mechanism by which the brain can enhance thalamocortical interactions in response to external stimuli [73, 74]. It is thus possible that both phenomena may contribute to the occurrence of fluctuations in DLB patients. Alternatively, thalamic functional disturbances could also be a consequence of cholinergic function deficits.

Overall, our results indicate that fluctuations in DLB patients may derive more directly from functional connectivity disturbances than from clear structural impairments. This is concordant with recent data supporting the hypothesis that DLB is a primary synaptopathy [75], in which the accumulation of phosphorylated α-synuclein in the form of small aggregates may cause synaptic dysfunction and loss that occur prior to Lewy body formation and neuronal loss and that are closely related to cognitive impairment.

This study has some potential limitations. First, DLB and AD patients were diagnosed based on clinical assessment, rather than a post-mortem pathological validation. However, diagnosis was made by experienced geriatricians, using standardized criteria with a high specificity confirmed by autopsy findings [76], and all participants were followed longitudinally. Second, fluctuations are a complex symptom encompassing attentional, cognitive, and alertness aspects that are mixed in the score obtained from the Mayo Fluctuation Scale, making the interpretation of the correlations with MRI measures more difficult. These results thus need to be treated with caution.

## Conclusions

Using both structural and functional MRI data, we found that functional connectivity changes, rather than significant gray matter loss, could play a role in the emergence of fluctuations in DLB patients. Notably, fluctuations in the DLB group appeared to be related to disturbances of the external functional connectivity of the salience network (responsible for switching between the default mode network and attentional executive networks), which may lead to less relevant transitions from one cognitive state to another in response to internal and environmental stimuli. Higher fluctuation scores were also related to a lower thalamic functional connectivity, suggesting that the thalamus could be a key region for the occurrence of this symptom. More globally, our results underline the interest of considering large-scale brain networks to investigate the neural bases of DLB core symptoms such as fluctuations.

## Supplementary information

Additional file 1: **Table S1.** MNI coordinates and anatomical labels of the regions of interest used in the functional connectivity analysis

Additional file 2: **Figure S1.** Pattern of voxel-wise gray matter loss in DLB patients compared with healthy elderly subjects at a more permissive significance threshold (*p*<0.0001 uncorrected for multiple comparisons). Abbreviations: BC: brainstem, Thal: thalamus

## Abbreviations

ACC: Anterior cingulate cortex
AD: Alzheimer’s disease
AI: Anterior insula
ANOVA: Analysis of variance
BOLD: Blood oxygen level-dependent
CDR: Clinical dementia rating
CM2R: Centre Mémoire de Ressources et de Recherche
CPP: Comité de protection des personnes
DAN: Dorsal attention network
DARTEL: Diffeomorphic anatomical registration through exponentiated lie algebra
DLB: Dementia with Lewy bodies
DMN: Default-mode network
FDR: False discovery rate
FEF: Frontal eye field
FPN: Frontoparietal network
FWE: Family-wise error
HC: Healthy controls
HUS: University Hospital of Strasbourg
ICA: Independent component analysis
IPS: Intraparietal sulcus
LP: Lateral parietal cortex
LPFC: Lateral prefrontal cortex
MNI: Montreal Neurological Institute
MPFC: Medial prefrontal cortex
MPRAGE: Magnetization-prepared rapid gradient-echo
MRI: Magnetic resonance imaging
PCC: Precuneal cortex
PPC: Posterior parietal cortex
ROI: Region of interest
RPFC: Rostral prefrontal cortex
SI: Substantia innominata
SMG: Supramarginal gyrus
SN: Salience network
SPECT: Single-photon emission computed tomography
SPM: Statistical Parametric Mapping
VBM: Voxel-based morphometry
