# Supplementary material for: Changes in gray matter volume and functional connectivity in dementia with Lewy bodies compared to Alzheimer’s disease and normal aging: implications for fluctuations
Source: Alzheimers Res Ther. 2020 Jan 6;12:9. doi: 10.1186/s13195-019-0575-z (PMC6945518; doi:10.1186/s13195-019-0575-z)
Supplement: Supplementary file 1 — Table S1. MNI coordinates and anatomical labels of the regions of interest used in the functional connectivity analysis [file 13195_2019_575_MOESM1_ESM.docx]

| Network | Anatomical region | MNI coordinates (x,y,z) | | |
| --- | --- | --- | --- | --- |
| Default-mode network | Medial prefrontal cortex | 1 | 55 | -3 |
|  | Left lateral parietal cortex | -39 | -77 | 33 |
|  | Right lateral parietal cortex | 47 | -67 | 29 |
|  | Precuneus | 1 | -61 | 38 |
| Salience network | Anterior cingulate cortex | 0 | 22 | 35 |
|  | Left anterior insula | -44 | 13 | 1 |
|  | Right anterior insula | 47 | 14 | 0 |
|  | Left rostral prefrontal cortex | -32 | 45 | 27 |
|  | Right rostral prefrontal cortex | 32 | 46 | 27 |
|  | Left supramarginal gyrus | -60 | -39 | 31 |
|  | Right supramarginal gyrus | 62 | -35 | 32 |
| Frontoparietal network | Left lateral prefrontal cortex | -43 | 33 | 28 |
|  | Right lateral prefrontal cortex | 41 | 38 | 30 |
|  | Left posterior parietal cortex | -46 | -58 | 49 |
|  | Right posterior parietal cortex | 52 | -52 | 45 |
| Dorsal attention | Left frontal eye field | -27 | -9 | 64 |
|  | Right frontal eye field | 30 | -6 | 64 |
|  | Left intraparietal sulcus | -39 | -43 | 52 |
|  | Right intraparietal sulcus | 39 | -42 | 54 |
| Additional Regions | Left thalamus | -10 | -19 | 6 |
|  | Right thalamus | 11 | -18 | 7 |
|  | Midbrain | 0 | -28 | -17 |

**Table S1. MNI coordinates and anatomical labels of the regions of interest used in the functional connectivity analysis.**
