# Supplementary figures and images for: Changes in gray matter volume and functional connectivity in dementia with Lewy bodies compared to Alzheimer’s disease and normal aging: implications for fluctuations
Source: Alzheimers Res Ther. 2020 Jan 6;12:9. doi: 10.1186/s13195-019-0575-z (PMC6945518; doi:10.1186/s13195-019-0575-z)

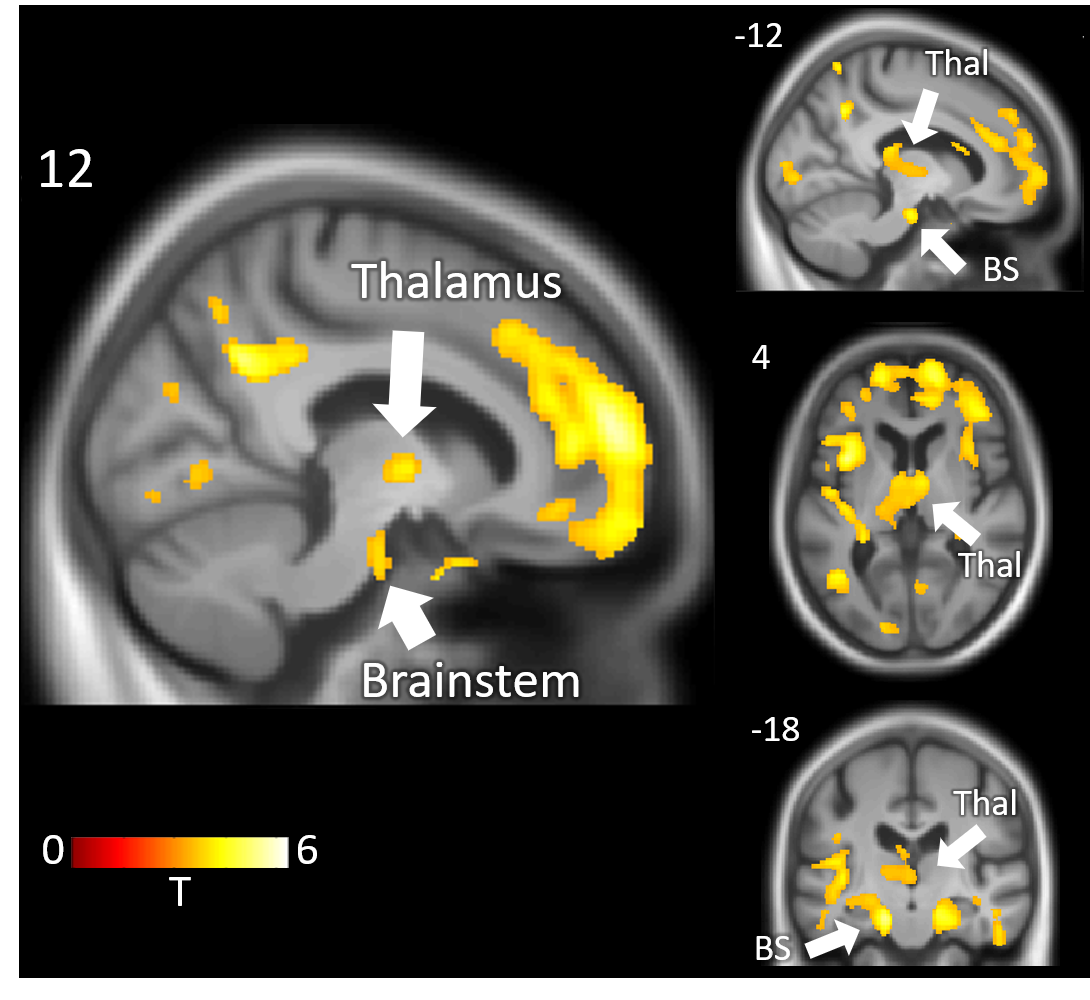

Supplement: Supplementary file 2 — Figure S1. Pattern of voxel-wise gray matter loss in DLB patients compared with healthy elderly subjects at a more permissive significance threshold (p<0.0001 uncorrected for multiple comparisons). Abbreviations: BC: brainstem, Thal: thalamus [file 13195_2019_575_MOESM2_ESM.tif]
